# Next-Generation Grade and Survival Expression Biomarkers of Human Gliomas Based on Algorithmically Reconstructed Molecular Pathways

**DOI:** 10.3390/ijms23137330

**Published:** 2022-06-30

**Authors:** Marianna A. Zolotovskaia, Max A. Kovalenko, Victor S. Tkachev, Alexander M. Simonov, Maxim I. Sorokin, Ella Kim, Denis V. Kuzmin, Betul Karademir-Yilmaz, Anton A. Buzdin

**Affiliations:** 1 Moscow Institute of Physics and Technology, 141701 Dolgoprudny, Russia; kovalenko.ma@phystech.edu (M.A.K.); simonov@oncobox.com (A.M.S.); sorokin@oncobox.com (M.I.S.); kuzmin.dv@mipt.ru (D.V.K.); 2 Omicsway Corp., Walnut, CA 91789, USA; tkachev@oncobox.com; 3 Laboratory of Clinical and Genomic Bioinformatics, I.M. Sechenov First Moscow State Medical University, 119991 Moscow, Russia; 4 Shemyakin-Ovchinnikov Institute of Bioorganic Chemistry, 117997 Moscow, Russia; buzdin@oncobox.com; 5 Clinic for Neurosurgery, Laboratory of Experimental Neurooncology, Johannes Gutenberg University Medical Centre, Langenbeckstrasse 1, 55124 Mainz, Germany; ella.kim@unimedizin-mainz.de; 6 Department of Biochemistry, School of Medicine/Genetic and Metabolic Diseases Research and Investigation Center (GEMHAM), Marmara University, Istanbul 34854, Turkey; betulkarademir@marmara.edu.tr; 7 World-Class Research Center “Digital Biodesign and Personalized Healthcare”, Sechenov First Moscow State Medical University, 119991 Moscow, Russia; 8 PathoBiology Group, European Organization for Research and Treatment of Cancer (EORTC), 1200 Brussels, Belgium

**Keywords:** human gliomas, glioblastoma, low grade gliomas, survival and prognosis biomarkers, RNA sequencing, gene expression, molecular pathway analysis, systems biology, interactome-assisted algorithms

## Abstract

In gliomas, expression of certain marker genes is strongly associated with survival and tumor type and often exceeds histological assessments. Using a human interactome model, we algorithmically reconstructed 7494 new-type molecular pathways that are centered each on an individual protein. Each single-gene expression and gene-centric pathway activation was tested as a survival and tumor grade biomarker in gliomas and their diagnostic subgroups (*IDH* mutant or wild type, *IDH* mutant with 1p/19q co-deletion, *MGMT* promoter methylated or unmethylated), including the three major molecular subtypes of glioblastoma (proneural, mesenchymal, classical). We used three datasets from The Cancer Genome Atlas and the Chinese Glioma Genome Atlas, which in total include 527 glioblastoma and 1097 low grade glioma profiles. We identified 2724 such gene and 2418 pathway survival biomarkers out of total 17,717 genes and 7494 pathways analyzed. We then assessed tumor grade and molecular subtype biomarkers and with the threshold of AUC > 0.7 identified 1322/982 gene biomarkers and 472/537 pathway biomarkers. This suggests roughly two times greater efficacy of the reconstructed pathway approach compared to gene biomarkers. Thus, we conclude that activation levels of algorithmically reconstructed gene-centric pathways are a potent class of new-generation diagnostic and prognostic biomarkers for gliomas.

## 1. Introduction

In gliomas, the expression of certain marker genes is strongly associated with survival, and in many studies, marker gene expression levels were better predictors of survival than histological assessments [1,2,3]. Moreover, expression-based models are widely used to classify gliomas into molecular subtypes, e.g., for a group of genes involved in angiogenesis [4]. Microarray gene expression clustering analysis revealed a diagnostic group of 170 genes that could classify gliomas into glioblastomas (GBM), lower grade astrocytomas, and oligodendrogliomas [5]. Furthermore, different expression-based models were built to predict survival of patients with gliomas: 21- [6], 44- [3], and 58-gene signatures [7]. Moreover, a 35-gene signature was created to classify high-grade gliomas into mesenchymal, proneural, and proliferative phenotypes—named by the similarities with the stages of neurogenesis [8].

Completion of large collections of RNA sequencing (RNA-seq) tumor profiles has strongly impacted the analysis of transcriptomic portraits of the gliomas. For example, the analysis of The Cancer Genome Atlas (TCGA) project database resulted in a new classification of GBM for proneural, neural, classical and mesenchymal subtypes [9]. Later on, validation of this classification model on 225 RNA-seq profiles obtained from another large data collection, the Chinese Glioma Genome Atlas (CGGA) project database, clearly confirmed signatures for proneural, neural, and mesenchymal subtypes, but not for the “classical” subtype [10]. However, later on, another study confirmed three subtypes of GBM: classical, pro-neural, and mesenchymal, where gene signatures were also correlated with the immune microenvironment [11]. The “missing” neural subtype might represent an artifact resulting from significant admixtures of non-tumor cells in the whole-tissue tumor specimens used for sequencing. Furthermore, an alternative transcriptome-based classification of GBM was proposed that had three different molecular subtypes called invasive, mitotic, and intermediate [12].

Besides protein-coding mRNAs, in many systematic transcriptomic studies of gliomas, attention was paid to non-coding RNAs, including long non-coding (lnc) RNAs. Expression of lncRNAs was found to correlate with malignancy grade and histological subtypes of gliomas in microarray datasets [13]. Later on, using Rembrandt and GSE16011, datasets three molecular glioma subtypes (LncR1-3) were revealed by distinct lncRNA signatures associated with patient survival [14]. The TCGA RNA-seq dataset then established a new standard for the analysis of lncRNAs for gliomas and normal brain tissues. For example, expressions of specific lncRNAs were specifically associated with the lower grade glioma (LGG) diagnostic genetic groups (classified by mutation in *IDH1/2* genes and by *1p19q* codeletion), and also with the classical, mesenchymal, neural, and proneural GBMs molecular subtypes. In total, 584 and 282 lncRNAs were associated with negative and positive survival prognosis, respectively [15]. The prognostic 8-lncRNA signature was created using TCGA and CGGA datasets to predict grade II and III glioma patients with poor outcomes [16].

Apart from genome-wide analyses of transcriptomic signatures, the relationships between single genes’ expression levels and clinical parameters have also been intensively investigated in gliomas. For example, expression of non-coding RNA *CRNDE* was found to be a robust negative predictor of survival and at the same time a strong diagnostic biomarker in gliomas [17].

Upregulated expression of gene for membrane type 1 metalloprotease (MT1-MMP) was associated with glioma invasion [18]. Expression of *HLA-DR* genes was associated with a more aggressive glioma tumor grade and shorter progression free survival (PFS) and overall survival (OS) times [19]. Deregulated expression of genes controlling circadian rhythms was specific for the high grade gliomas [20]. The meta-analysis of 21 studies, including 1322 glioma patients, showed that *TP53* expression was associated with survival in gliomas [21].

On the other hand, the expression of *CD44* was associated with the histological grade but not with survival [22]. Some members of cancer/testis antigens (*CTCFL* and *ACTL8*) have been associated with the poorer prognosis in glioma patients [23]. High expression of *ABCC8* mRNA is associated with longer overall survival [24]. The overexpression of *CRM1* negatively correlated with glioma grade and was associated with poor prognosis [25].

Both single-gene-centered and genome-wide profiling have generated a wealth of information on glioma-associated transcriptomic landscapes and enabled the development of computational models for glioma progression basing on gene expression data. Application of integrative bioinformatics to analyze the data from transcriptomic analyses of glioblastoma tissues and glioblastoma stem cells have revealed that profound changes in the activity of clinically relevant signaling pathways can result from the cumulative impacts of multiple genes that may show only modest changes in their expression (Kim et al. Cancers 2020). Further underscoring this notion are our recent findings related to *FREM2* and *SPRY1* genes: they are associated with negative prognosis and more aggressive histological types of IDH-WT glioblastomas [26]. We have established that an algorithmically reconstructed molecular pathway centered around FREM2 predicts negative associations with survival and molecular and histological subtypes of gliomas considerably more strongly than the paternal gene FREM2 alone (Zolotovskaia et al., 2021). These findings merited in-depth further validation in a larger study that would sufficiently cover the spectrum of glioma-associated genes.

In this study, we used a human interactome model containing 64,095 participants and 361,654 molecular interactions. We algorithmically reconstructed 7494 new-type molecular pathways that are centered each on an individual specific protein. We then compared biomarker capacities of single-gene expression profiles with activation levels of these algorithmically deduced gene-centric pathways in human gliomas. To this end, we took RNA sequencing profiles annotated with survival data from the glioma databases of The Cancer Genome Atlas (TCGA) and the Chinese Glioma Genome Atlas (CGGA) projects, including, in total, 527 glioblastoma (GBM) and 1097 low grade glioma (LGG) profiles. Each gene or pathway was tested as a survival and tumor grade biomarker in the gliomas and in their diagnostic subgroups (*IDH* mutant or wild type, *IDH* mutant with 1p/19q co-deletion, *MGMT* promoter methylated or unmethylated), and also in the three specific GBM molecular subtypes (proneural, mesenchymal, classical). With adjusted *p*-value 0.05 as the threshold, we identified 2724 such gene and 2418 pathway survival biomarkers out of total 17,717 genes and 7494 pathways investigated. Thus, the pathway approach was about two times more effective than the assessment of single genes as survival biomarkers. We then assessed tumor grade and molecular subtype biomarkers, and with the threshold of AUC > 0.7 identified 1322/982 and 472/537 gene/pathway biomarkers, respectively. This suggested about 1.9 and 2.6 times, respectively, greater relative efficacy of the reconstructed pathway approach. Thus, we conclude that activation levels of algorithmically reconstructed gene-centric pathways are a potent class of new-generation diagnostic and prognostic biomarkers for gliomas.

## 2. Results

### 2.1. Generation of Gene-Centric Molecular Pathways

We used the human interactome model [27] to algorithmically generate gene-centric molecular pathways. Overall, the interactome model is a directed graph, where nodes are genes or metabolites, and edges are known pairwise molecular interactions. It incorporates molecular participants and interactions extracted from 51,672 source molecular pathways, in total. To build the model, each pathway graph was processed as follows: pathway nodes containing *n* molecular participants were divided into *n* nodes with only one participant, and the corresponding interactions were assigned between such single nodes. Biochemical reactions and transport processes were processed as auxiliary nodes, when involving more than two participants. We then combined together, all pathway graphs were based on the coinciding unique gene products and/or metabolites.

Molecular participants which were not connected with the overall network were excluded (excluding less than 1% of the initial molecular participants). The remaining interactors formed a connected graph including 122,929 vertices and 600,137 edges. In total, it incorporated products of 7496 protein coding genes, 56,599 other molecular participants, and 361,654 interactions between them (excluding auxiliary nodes and interactions). The graph density was 8.08 × 10^−5^, and average vertex degree (the number of edges connecting the vertex) was 4.9.

**Figure 1:**
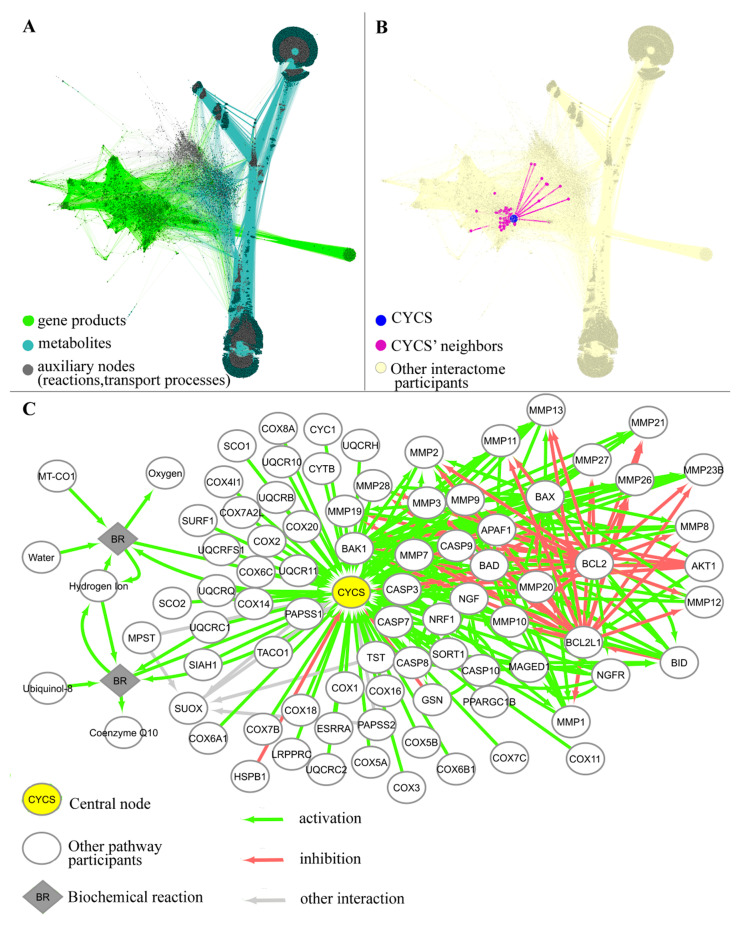
(**A**) Integrated human interactome model (adapted from [27]) combining gene products and metabolites. Edges inherit color of the corresponding nodes. (**B**) Example of gene-centric pathway for gene *CYCS*, which serves as the central node (enlarged blue dot), and its direct interactors are the pathway participants (violet dots). Edges between the central node and its interactors are shown in violet. (**C**) Detailed scheme of *CYCS* gene-centric pathway, where CYCS is the central node, and its direct interactors are the pathway participants. Colors of edges denote types of interaction (inhibition, activation, other).

A neighborhood of each gene product was selected as a gene-centric molecular pathway. A neighbor was considered an interactome vertex which was directly connected with the gene product by one edge. If neighbor was a “process” node (biochemical reaction or transport process), then all participants of this process would become neighbors to preserve integrity of the reaction or transport process. Thus, each gene-centric pathway contained a central node gene product, and its direct interactants and fellow participants in biochemical reactions or transport processes in which central gene products are involved, as exemplified in Figure 1.

In this way, we obtained 7496 gene-centric molecular pathways. Then, the pathways were algorithmically functionally annotated as described previously [28], and we assigned activator/repressor role coefficients (ARRs) to each pathway node. An ARR value characterizes functional role of a gene product in the pathway and takes value of −1 when a gene product inhibits the pathway; 1 when it activates the pathway; 0 when it has an neutral or unclear role in the pathway; 0.5 or −0.5, when it activates or inhibits the pathway, respectively.

**Figure 2:**
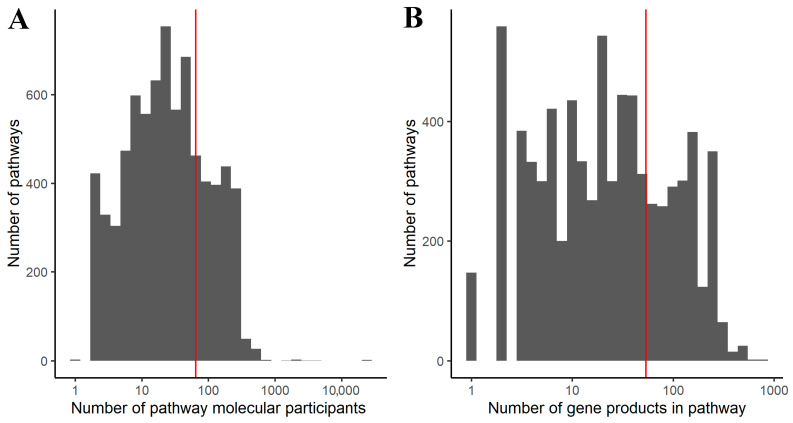
Size distribution of gene-centric molecular pathways (**A**) when all molecular interactors are considered; (**B**) when only gene products are considered. Vertical red line shows mean a value.

In terms of gene products, the sizes of such pathways varied from 1 to 777, and mean pathway size was 22 (Figure 2). Two pathways were excluded from further analysis because all assigned ARR coefficients for their members were equal to zero.

### 2.2. Single-Gene-Expression Biomarkers as Overall Survival Predictors in Gliomas

RNA-seq profiles from one TCGA and two CGGA datasets have different numbers of genes characterized by expression levels. There are only 17,717 genes common to all databases. We used univariate Cox analysis and the log-rank test to analyze the expression of each gene in relation to the available overall survival (OS) data. To this end, the patients were categorized relatively to the median value of each gene expression level. Hazard ratio (HR) and FDR-adjusted (Benjamini–Hochberg) *p*-value for Kaplan–Meyer analysis (log-rank test) were obtained for each gene product. A particular gene product was considered a significant survival biomarker if it had an FDR-adjusted *p*-value < 0.05 in all three datasets under analysis, and a consistent direction of expressional change (either up- or downregulation linked with survival in all three datasets).

In both GBM and LGG, we also separately analyzed the genetically characterized subgroups with *IDH* mutation, with *IDH* mutation and 1p/19q codeletion, with wild type (wt) *IDH*, and with methylated or unmethylated *MGMT* promoter.

**Figure 3:**
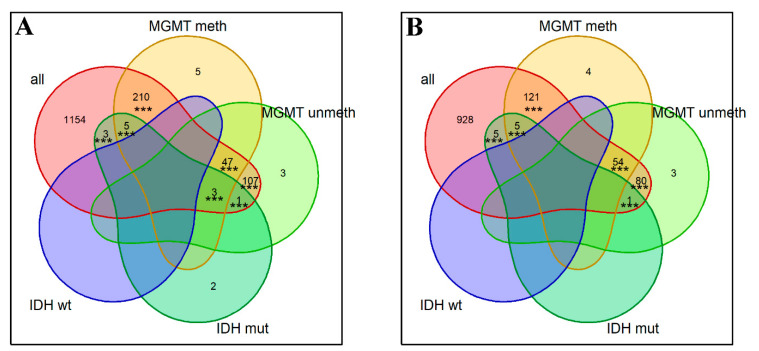
Numbers of gene expression biomarkers significantly associated with (**A**) shorter OS or (**B**) longer OS in the different LGG groups. Asterisks show significance levels of permutation testing for the respective intersections: *** for *p* < 0.001.

We did not detect any genes meeting the above OS prognosis biomarker criteria in GBM and in GBM molecular subgroups. No biomarkers were found for the LGG subgroup with 1p/19q codeletion or for the wt *IDH* subgroup. However, for the whole LGG cohort, we found as many as 2724 biomarker genes that were statistically significantly associated with OS (permutation test *p* < 10^−5^ for intersection of biomarkers from three datasets: TCGA, CGGA_325, CGGA_693); see Supplementary Table S1. We also detected a number of OS biomarkers in the three molecular subgroups of LGG: 454 genes in *MGMT*-methylated (*p* < 10^−5^), 299 in *MGMT*-unmethylated (*p* < 10^−5^), and 25 in *IDH* mutant LGGs (*p* < 10^−5^) (Figure 3, Supplementary Table S1). Among them, there were comparable numbers of positive and negative prognosis biomarkers (Figure 3). Of note, many marker genes intersected with all LGG subgroups, and most of these intersections were non-random according to permutation testing (Figure 3).

**Figure 4:**
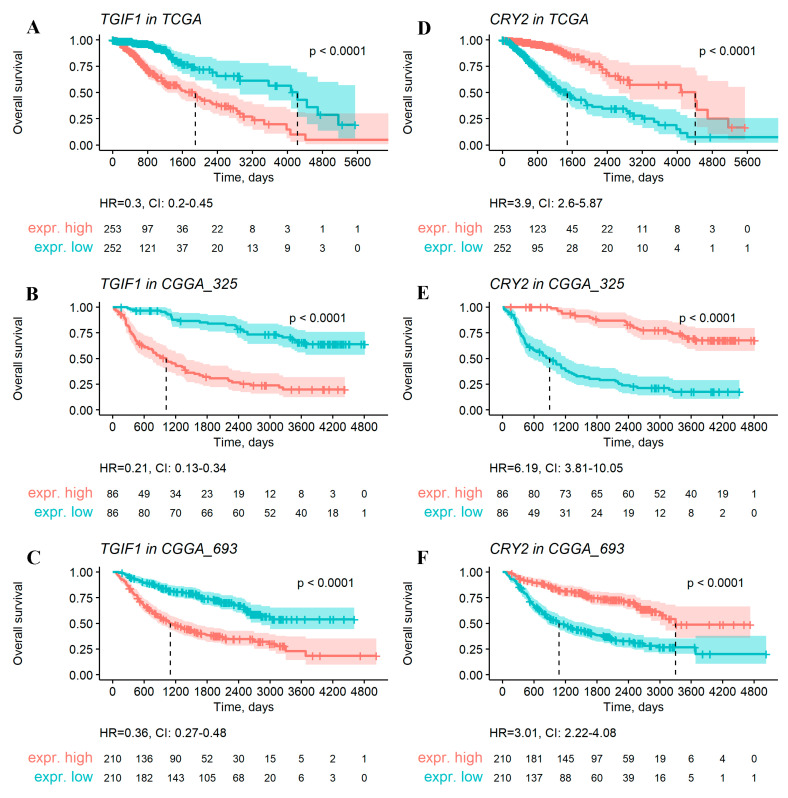
Examples of the negative (*TGIF1* gene, panels (**A**–**C**)) and positive (*CRY2* gene, panels (**D**–**F**)) overall survival gene expression biomarkers identified using a systematic statistical approach on LGG data from TCGA (**A**,**D**), CGGA_325 (**B**,**E**), and CGGA_693 (**C**,**F**) datasets. “High” and “low” levels were defined relative to a median gene expression value. Hazard ratio (HR) and its confidence interval (CI) is shown for every analysis.

Examples of two such LGG positive and negative OS biomarker genes are shown in Figure 4.

**Figure 5:**
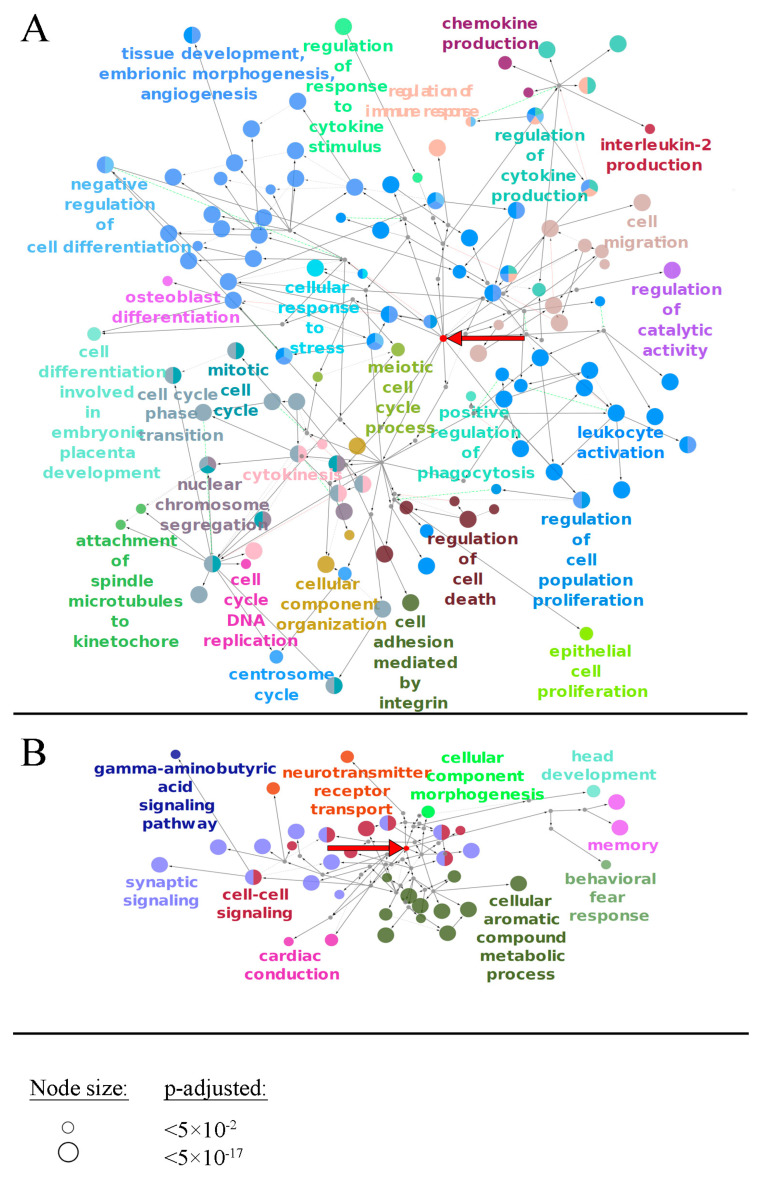
Gene Ontology tree (from null to third level) for genes linked with negative (**A**) and positive (**B**) prognoses in LGG. Bold red arrow shows a root node (null level of GO tree). Small grey nodes are parental nodes connecting the root node with significant terms of the third level. The size of the node reflects its adjusted *p*-value. Each group of terms is marked by a specific color; if a term belongs to several groups, it has several colors, accordingly.

We then performed Gene Ontology (GO) analysis for the discovered OS biomarker genes of *MGMT*-methylated, *MGMT*-unmethylated, *IDH* mutant, and total LGGs, separately for the positive and negative prognosis gene expression biomarkers. Significantly enriched terms (adjusted *p* < 0.05) for the first to third levels of GO tree were visualized on Figure 5 and Figure S1. GO terms enriched for all levels of the GO tree (adjusted *p* < 0.05) are listed in Supplementary Table S2.

In the total LGG group, we observed the trend that the biological processes (BPs) linked with specific aspects of neuronal functioning and neuronal cell differentiation prevailed for the genes associated with the favorable prognosis. In contrast, BPs related to embryonal development, cell cycle progression, remodeling of the immune system, regulation of cell death, and integrin-mediated adhesion were specific for the gene biomarkers associated with poor prognosis (Figure 5).

### 2.3. Gene-Centric Molecular Pathways as Overall Survival Predictors for Gliomas

**Figure 6:**
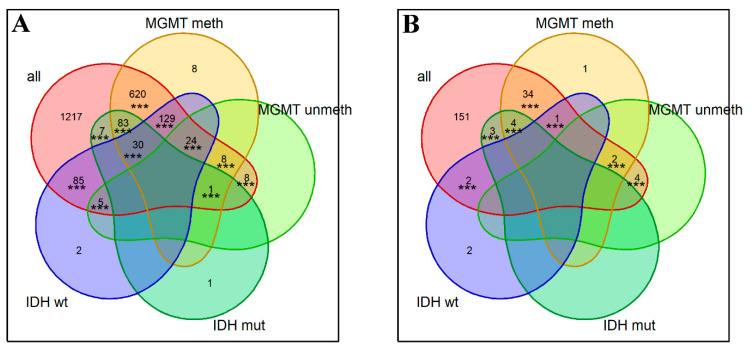
Numbers of gene-centric pathway biomarkers significantly associated with (**A**) shorter OS and (**B**) longer OS in the different LGG groups. Asterisks show significance levels of permutation testing for the respective intersections: *** for *p* < 0.001.

Analogously, we then tested all gene-centric pathways as the OS biomarkers in glioma patients. For every pathway, we investigated whether its pathway activation level (PAL) values were associated with OS in the three datasets under analysis. The same statistical thresholds and settings were applied as for the individual genes in the above section. Similarly to the individual gene assay, no survival biomarkers were found for GBM and all of its subgroups. However, we detected 2418 gene-centric pathways in total, whose PAL values served as the robust OS biomarkers for LGG. In addition, we found biomarker pathways for all four LGG subgroups (in contrast to the single-gene biomarker assay, where only three LGG subgroups had biomarker genes). Among them, 903 biomarker pathways were found for *MGMT*-methylated (*p* < 10^−5^), 52 for *MGMT*-unmethylated (*p* < 10^−5^), 129 for *IDH*-mutant (*p* < 10^−5^), and 280 for wt *IDH* (*p* < 10^−5^) LGGs (Figure 6, Supplementary Table S3).

**Figure 7:**
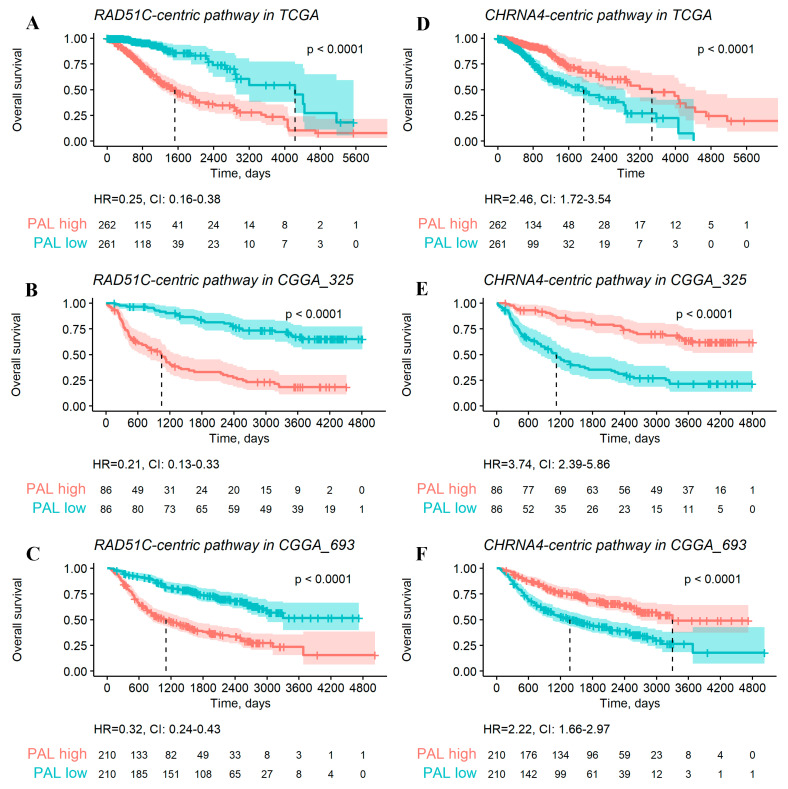
Examples of the negative (*RAD51C* pathway, panels (**A**–**C**)) and positive (*CHRNA4* pathway, panels (**D**–**F**)) OS gene-centric pathway biomarkers of LGG identified for TCGA (**A**,**D**), CGGA_325 (**B**,**E**), and CGGA_693 (**C**,**F**) datasets. “High” and “low” levels were defined relative to a median pathway activation level. Hazard ratio (HR) and its confidence interval (CI) are shown for every analysis.

**Figure 8:**
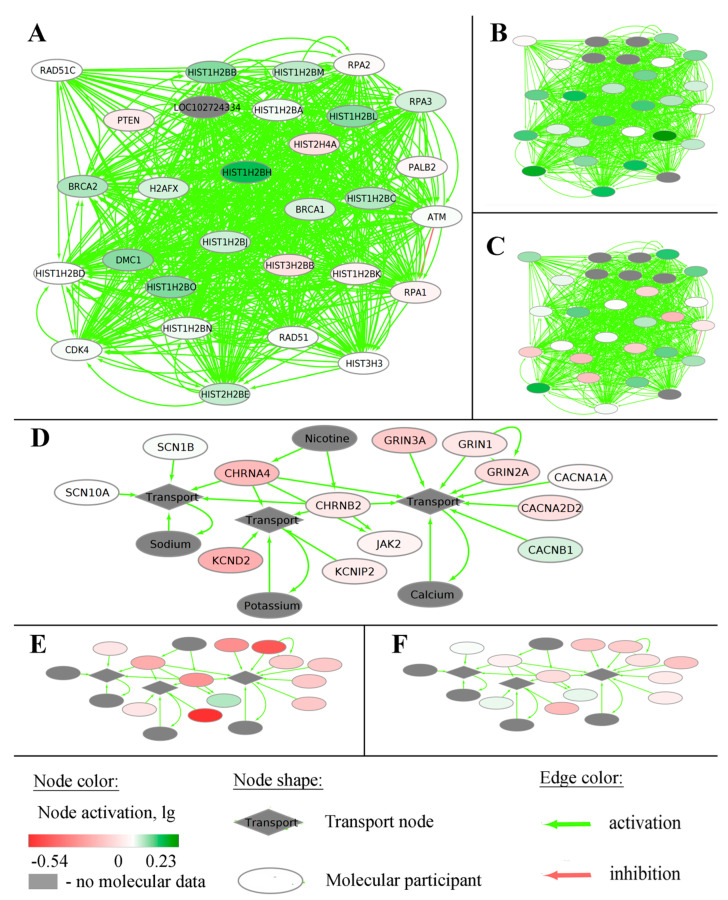
Pathway activation charts of the gene-centric molecular pathways taken as examples. of negative and positive OS biomarkers in LGG. The *RAD51C*-centric pathway that is linked with negative prognosis shown for averaged (**A**) TCGA, (**B**) CGGA_325, and (**C**) CGGA_693 LGG expression datasets. Nodes on panel A correspond to nodes on panels B and C. The *CHRNA4*-centric pathway that is linked with positive prognosis is shown for averaged (**D**) TCGA, € CGGA_325, and (**F**) CGGA_693 LGG expression datasets. Nodes on panel (**D**) correspond to nodes on panels (**E**,**F**). Node activation is a logarithmic ratio of the geometric mean of expression in patients with negative prognoses to the geometric mean of expression in patients with positive prognoses.

Examples of such negative (*RAD51C-pathway*) and positive (*CHRNA4-pathway*) OS biomarkers for total LGG cohort are shown in Figure 7. The pathway activation chart is shown in Figure 8, where activation of each node corresponds to the ratio of the geometric mean of expression in LGG patients with negative prognoses to the geometric mean of expression in LGG patients with positive prognoses.

We then attempted to functionally characterize the biomarker gene-centric pathways by GO analysis. Each gene-centric pathway was considered as a set of gene products, and enrichment values were calculated for those sets against the control of all protein-coding human genes in the GO database. Significantly enriched GO terms (adjusted *p* < 0.05) were interrogated for every pathway under study. We considered that a “functional group” is the set of pathways with the same GO tag, e.g., “cytokinesis.” A pathway may be included in several functional groups if it has several enriched GO tags. For the negative prognosis biomarker pathways, we identified 6158 such GO groups for all LGGs, 3533 for *IDH*-mutated, 3841 for wt *IDH*, 5818 for *MGMT* methylated, and 2598 for *MGMT* unmethylated LGGs.

**Figure 9:**
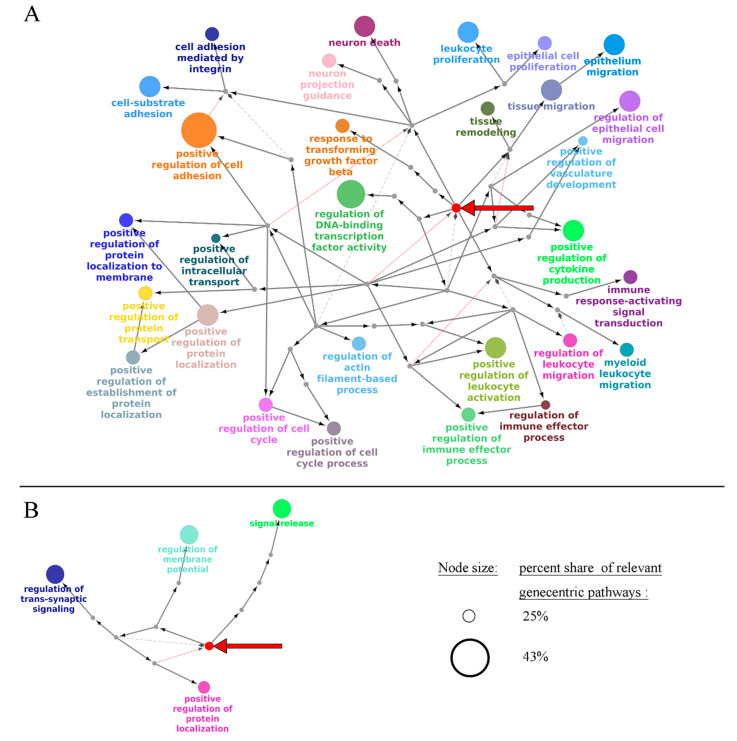
Gene Ontology tree (from null to third level) for gene-centric pathways linked with negative (**A**) and positive (**B**) prognoses in LGG. Bold red arrow shows a root node (null level of GO tree). Small grey nodes are parental nodes connecting a root node with significant terms of the third level. The size of a node reflects its adjusted *p*-value. Each group of terms is marked by a specific color.

For the positive prognosis biomarker pathways, there were 5263 GO groups for all LGGs, 447 for *IDH*-mutated, 788 for wt *IDH*, 2917 for *MGMT* methylated, and 416 for *MGMT* unmethylated LGGs; see Supplementary Table S4. We then explored the biggest functional groups (containing 25% of pathways or more) by grouping them into a GO-tree using Cytoscape + CluePedia, and then visualized the first three levels of the tree obtained (Figure 9 and Figures S1–S8).

Overall, we observed a largely similar landscape of GO terms associated with the negative/positive OS as for the above biomarker gene assay (Figure 5 and Figure 9). For the positive prognosis biomarkers, we detected the biological processes (BPs) linked with specific neuronal functions, whereas for the negative prognosis they were related to embryonal tissue development, cell cycle progression, immune remodeling, cell adhesion, protein transport, and membrane localization.

### 2.4. Performances of Single Gene and Gene-Centric Pathway Biomarkers

We then compared the performances of single-gene biomarkers and of gene-centric pathway biomarkers. For the total of 6467 gene-centric pathways, in the previous test we identified 3782 biomarker cases. For the total of 17,717 individual genes, we found 3505 biomarker cases. Thus, the relative biomarker capacity in such test was about three times higher for the gene-centric pathways.

**Figure 10:**
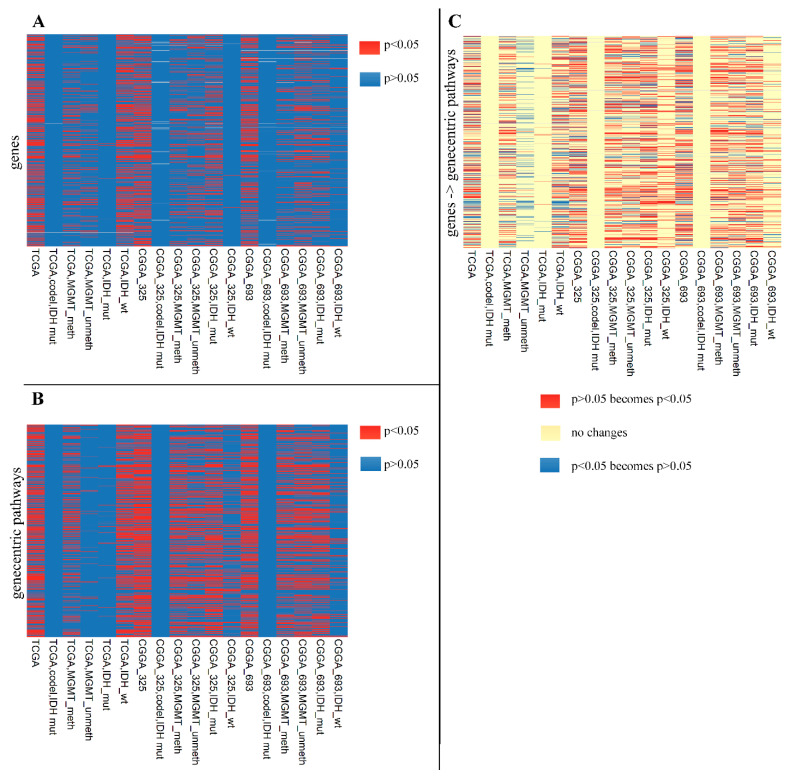
Adjusted log-rank test *p*-values for (**A**) 6467 individual genes as OS biomarkers in LGG and its subtypes; (**B**) 6467 gene-centric pathways as OS biomarkers in LGG and its subtypes. (**C**) Comparison of log rank test adjusted *p*-values when comparing a gene as the OS biomarker and the corresponding gene-centric pathway as the OS biomarker; here red bands mean better performance of pathways, and blue bands mean better performance of single-gene biomarkers.

We also compared the biomarker capacities in a different way. For these estimates, we considered that “case” is OS analysis for one potential biomarker (gene or pathway) in only one dataset (TCGA, CGGA_325, or CGGA_693). “Significant” cases were defined as those having log-rank test adjusted *p*-values < 0.05. In total, we identified 20,507 significant cases on the gene level (Figure 10A) and 32,995 cases on the pathway level (Figure 10B). Considering identical sizes for starting sets on the gene and pathway “teams” (6467 each), we concluded that the gene-centric pathways were overall more effective as biomarkers because they gave ~61% more biomarker cases than the single genes.

In pairwise comparisons, we also compared biomarker capacities of the genes and of the respective gene-centric pathways. For example, *PTCH1* was significant in one comparison, and the PTCH1-centric pathway was significant in 14 comparisons. In total, 3856 gene-centric pathways showed better biomarker capacities than the corresponding single genes, versus only 1803 single genes which appeared as better biomarkers than the gene-centric pathways; 673 genes and the corresponding pathways demonstrated identical performance. Thus, the overall biomarker performance of gene-centric pathways in the latter test was ≈2.1 times greater than that of the single genes.

Thus, in all the comparisons performed we detected better OS biomarker capacity for the gene-centric pathways than for individual genes.

### 2.5. Individual-Gene and Gene-Centric Pathways as the Histological and Molecular Type Biomarkers

We then investigated how gene and pathway biomarkers can distinguish between histological and molecular types of the gliomas.

Using AUC > 0.7, a *t*-test adjusted *p*-value < 0.05 and the same directions of change in each dataset as statistical settings, the logarithmic expression levels of 17,717 genes and PAL values for 7494 gene-centric pathways were tested for their capacities to discriminate samples between LGG and GBM. First, 740 and 582 (*p* < 10^−5^) up- and downregulated differential genes were detected in GBM in comparison to LGG, respectively. In parallel, using the same statistical settings as for the above test, we detected 794 and 188 (*p* < 10^−5^) up- and downregulated pathway biomarkers, respectively (Supplementary Table S5).

**Figure 11:**
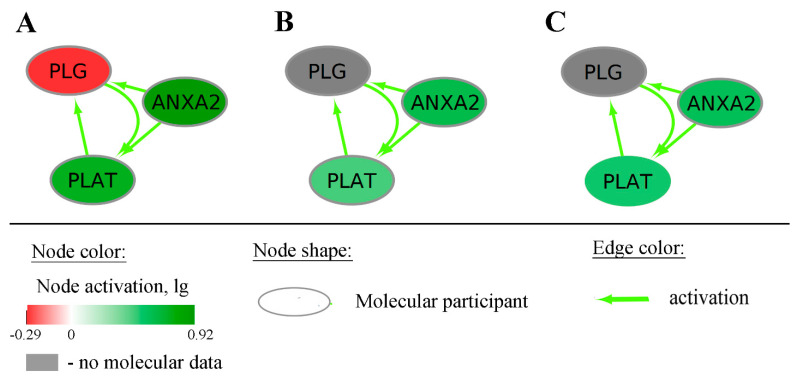
Pathway activation chart of the *ANXA2*-centric pathway based on TCGA (**A**), CGGA_325 (**B**), and CGGA_693 (**C**) datasets. Node activation was calculated as a logarithmic ratio of the geometric mean of expression levels in GBM patients to the geometric mean of expression levels in LGG patients.

We identified 26 genes and 21 pathways which had AUC >0.8 in all three datasets under study and were the best biomarkers in this GBM–LGG comparison (Table 1). Remarkably, among the best biomarkers, there was the gene *ANXA2* and also the corresponding gene-centric pathway, both upregulated in GBM compared to LGG (Figure 11). At the time of writing this manuscript, there were in total 17 scientific publications in PubMed crosslinking *ANXA2* with GBM (https://pubmed.ncbi.nlm.nih.gov/?term=anxa2+glioblastoma, date of access 28 May 2022). We hypothesize here that its gene-centric pathway is an important addition to reconstituting the overall GBM-specific interactome network.

We then attempted to identify gene- and pathway biomarkers which can differentiate the three GBM molecular subtypes (mesenchymal, classical, proneural) according to [11]. We performed pairwise comparisons between these classes and found comparable sets of biomarkers for both genes and gene-centric pathways, using AUC > 0.7, *t*-test adjusted *p*-value < 0.05, and the same directions of change in each dataset (Table 2 and Table S5). The best of these biomarkers (AUC > 0.8 in each dataset) are shown on Table 3. Notably, an outstandingly low number of biomarkers (four at the gene level and nothing at the pathway level) were detected in the comparison of the classical and proneural phenotypes (Table 2). This is in line with the unclear status of “proneural” group, which is frequently considered to arise from a mixture of a “classical” tumor with adjacent normal tissues in a sequenced biosample.

**Figure 12:**
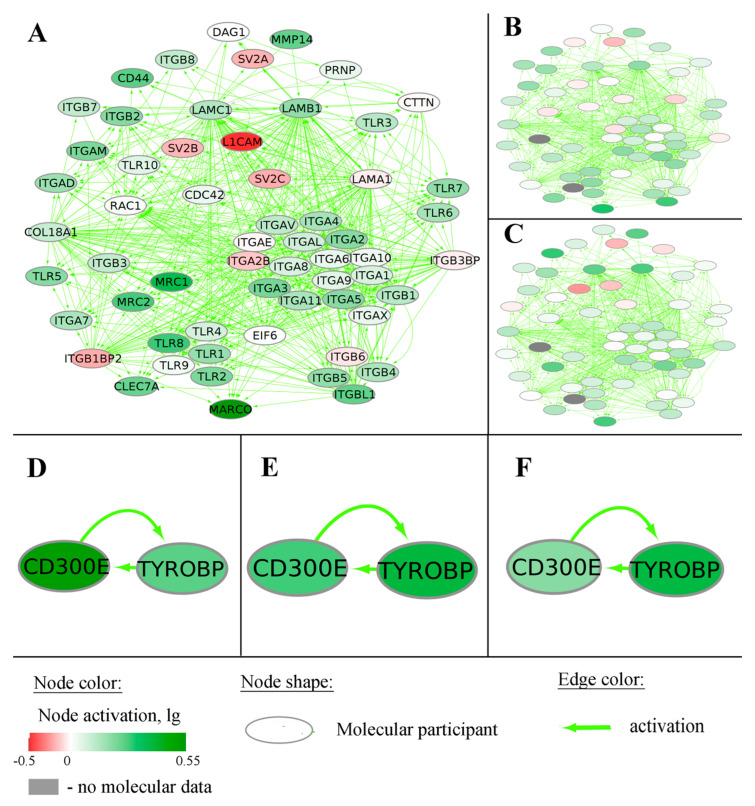
Pathway activation charts for marker pathways in mesenchymal vs. proneural GBM molecular subtypes. LAMB1-centric pathway: TCGA (**A**), CGGA_325 (**B**), and CGGA_693 (**C**) expression datasets. Nodes on panel A correspond to nodes on panels B and C. CD300E-centric pathway, TCGA (**D**), CGGA_325 (**E**), CGGA_693 (**F**) expression datasets. Node activation was calculated as a logarithmic ratio of geometric mean of expression levels in the GBM subgroups under analysis.

For the comparison of mesenchymal and proneural phenotypes, 32 genes and 283 gene-centric pathways were detected as the biomarkers (Table 2 and Table S5). Among them were five genes and their corresponding marker gene-centric pathways—*COL5A1*, *IL1R1*, *IL7R*, *LAMB1*, and *PLAUR*—which were up-regulated in the mesenchymal phenotype. The pathway activation chart for one of these pathways (*LAMB1*-centric pathway) is shown as an example in Figure 12. Mean AUC was slightly lower for *COL5A1*, *IL1R1*, and *LAMB1* gene expression than for the corresponding gene-centric pathways (0.8 vs. 0.81, 0.77 vs. 0.78, 0.75 vs. 0.78, respectively). The inverse picture was found for *IL7R* and *PLAUR* and their corresponding pathways (mean AUC 0.79 vs. 0.78, 0.8 vs. 0.77, respectively).

At the time of writing this manuscript, *LAMB1* was claimed as a key survival biomarker in mesenchymal glioblastoma [29]. *COL5A1* was described as biomarker of poor prognosis, promoting glioblastoma progression via the *PPRC1-ESM1* axis, and a mesenchymal-subtype-related gene [30,31,32]. *IL1R1* was shown to be linked with brain edema and tumor progression in mice [33,34]. While IL7R was not described as associated with glioblastoma pathogenesis or molecular subtype, there are 51 PubMed articles linking PLAUR and glioblastoma (https://pubmed.ncbi.nlm.nih.gov/?term=plaur+glioblastoma, date of access 28 May 2022). We suggest that the corresponding gene-centric pathways for these five genes may be important additions in understanding mesenchymal glioblastoma pathogenesis.

For the comparison of mesenchymal and classical GBM phenotypes, 440 genes and 254 gene-centric pathways met statistical criteria of AUC > 0.7, *t*-test adjusted *p*-value < 0.05, and the same directions of change in each dataset (Table 2 and Table S5). However, only 15 genes and four gene-centric pathways showed AUC > 0.8 in all datasets (Table 3). Interestingly, for CD300E-centric pathway (Figure 12), the central gene of a pathway was also a significant biomarker yet having lower AUC (0.76 for gene vs. 0.83 for pathway). Both such biomarkers were upregulated in mesenchymal compared to classical phenotype. Previously, *CD300E* was detected as the receptor stimulating monocyte activity and was even suggested as a potential antigen for an RNA vaccine against GBM [35,36].

**Figure 13:**
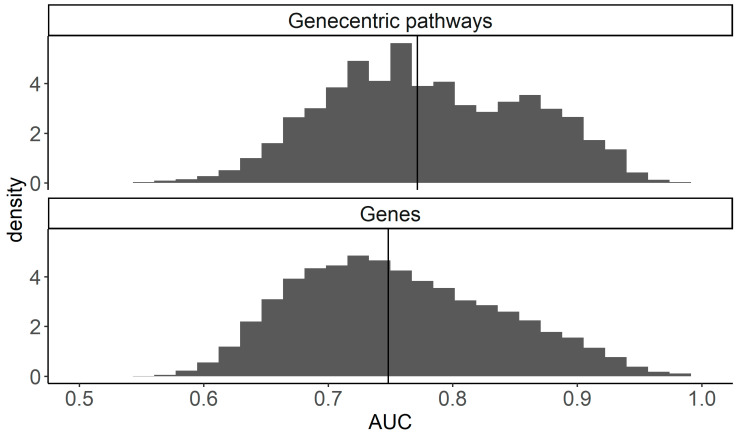
Distribution of maximal AUC values for expression-based biomarkers in the comparisons of GBM vs. LGG, Mesenchymal vs. proneural, classical vs. proneural, and mesenchymal vs. classical GBM molecular subtypes. The median of each distribution is shown by a marker line.

Finally, we compared the total distributions of AUC values for the gene- and gene-centric pathway biomarkers. To this end, the maximal AUC was assigned for each gene or pathway among those obtained in all of the above comparisons (LGG vs. GBM, mesenchymal vs. proneural GMB, classical vs. proneural GBM, mesenchymal vs. classical GBM). We found that the overall median of AUCs for the gene-centric pathways was higher than for the individual gene expression biomarkers: 0.771 vs. 0.748, respectively (Figure 13). Thus, in the molecular phenotype comparisons, the gene-centric pathways also showed themselves as the superior class of gene expression glioma biomarkers.

## 3. Discussion

We report here 7494 algorithmically generated molecular pathways centered each around a specific gene in a human interactome model. We tested the transcriptional activation levels of such next-generation pathways as survival and grade biomarkers in human gliomas. In parallel, we similarly tested the expression levels of 17,717 individual genes as biomarkers.

We used to our knowledge the largest clinically annotated public glioma RNA-seq collections as the testing dataset. We set relatively strict criteria for the survival biomarkers in all three expression datasets under analysis, which appeared challenging for some previously published glioma biomarkers, such as *CRNDE* [17] and *FREM2* [26,37] genes, which did not pass these conditions.

In both classes of genes and gene-centric pathways, we found no high-quality biomarkers for overall survival in GBM. The absence of clear genes and pathway biomarkers observed here may point to compromised biomarker capacity for previous expression biomarkers (such as *EGFR* and *PD-L1*), which may explain the controversial results for them in previous studies [38]. This may reflect outstandingly high heterogeneity in this group of tumors—at both the intra- and intertumoral levels [39,40]. Different sites within one GBM tumor may have different active driver fusion oncogenes [41], which complicates the molecular diagnosis and finding of effective long-standing treatment.

However, we identified a number of high-quality survival biomarkers for LGG and its genetic subtypes: 2724 for the gene and 2418 for the pathway biomarkers. Although there were a slightly larger number of gene-based survival biomarkers, their percentage share was about half that of the gene-centric pathways: 15.4% vs. 32.3%, respectively.

Interestingly, although the numbers of gene and pathway biomarkers of survival were comparable for both positive and negative prognoses in LGGs, we detected a much higher number of enriched GO terms for the pool of negative prognostic biomarkers—on both gene and pathway levels. While a positive OS prognosis in LGG was predominantly linked with the GO terms dealing with the normal CNS functioning and neural cell differentiation, a negative prognosis was characterized by a more numerous and diverse group of processes. These included cell cycle progression, proliferation, cell adhesion, embryonal development, DNA repair and damage checkpoints, and modulation of the inflammatory and immune responses.

In addition, we detected several cases when both single genes and their specific pathways acted as robust biomarkers in our model investigation (e.g., for genes *ANXA2*, *CD300E*, and *LAMB1*). We therefore hypothesize that considering such gene-centric pathways may be useful for building integrated gene networks involved in various aspects of molecular pathogenesis of human gliomas.

We also compared the performances of gene and gene-centric pathways as the expression biomarkers for the comparisons of GBM vs. LGG, and for the different GBM molecular types. In all the comparisons, we detected better capacity for the gene-centric pathways. This was also reflected by overall higher median maximal AUC for the biomarker assessment: 0.771 for the pathways vs. 0.748 for the individual genes. This evidently superior performance of the pathways as expression biomarkers may be a projection of a more general figure of the greater stability and robustness of pathway-level data compared to the gene-level data, as reported for the analysis of both transcriptomic [42,43] and proteomic [44] high-throughput expression profiles.

Finally, we conclude that activation levels of algorithmically deduced gene-centric molecular pathways are a potent class of next-generation diagnostic and prognostic biomarkers for human gliomas. Their applicability to other diseases, non-pathological conditions, and biological processes will be a matter of further study.

## 4. Materials and Methods

### 4.1. The Cancer Genome Atlas (TCGA) Dataset

Overall survival data were extracted from clinical annotations on gdc portal for 591 GBM and 510 LGG samples [45]. RNA-seq data (HTseq counts) were downloaded from gdc portal [45]. Only primary tumor samples with RNA data were selected (153 and 505 samples, respectively). *MGMT* methylation statuses were taken for GBM and LGG samples, respectively, from the reports [46] and [47]. *IDH* mutation statuses were extracted for GBM and LGG samples from SNV data (vcf files) from the gdc portal.

### 4.2. The Chinese Glioma Genome Atlas Dataset CGGA_325

Overall survival data, *IDH* mutation status, *MGMT* methylation status, and patient age information were extracted from clinical description for 137 GBM and 172 LGG samples from CGGA database; dataset id: mRNA-seq_325 [48,49]. RNA-seq data (RSEM counts) were downloaded for the corresponding biosamples.

### 4.3. The Chinese Glioma Genome Atlas Dataset CGGA_693

Overall survival data, *IDH* mutation status, *MGMT* methylation status, and patient age information were extracted from clinical description for 237 GBM and 420 LGG samples included in CGGA database; dataset id: mRNA-seq_693 [50,51]. RNA-seq data were downloaded for the corresponding biosamples.

### 4.4. Glioma Classification and Diagnosis

We used histological and WHO grade classifications that were available at the date of sample publication and annotation, and that were available in the corresponding original research papers. GBM molecular subtype classification results were obtained from GlioVis portal for TCGA and CGGA datasets [52]; see Table 4.

### 4.5. Source Molecular Pathways

The gene composition and molecular architectures of 51,672 intracellular pathways were extracted from the publicly available databases Reactome [53], NCI Pathway Interaction Database [54], Biocarta [55], HumanCyc [56], Qiagen [57], PathBank [58], and Oncobox database [28]; and processed as described in [28].

### 4.6. Pathway Activation Level Calculation

Pathway activation level (PAL) is an integral quantitative and qualitative characteristic of changes in the expression levels for genes participating in a certain molecular pathway [28,59,60]. PALs were calculated as follows:

$$PAL_{p}=\sum_{n}ARR_{np}*\mathrm{lg}\left(CNR_{n}\right)/\sum_{n}|ARR_{np}|\;*\;100,$$

where *PAL_p_* is *PAL* for pathway *p*; *CNR_n_* is *case-to-normal ratio*, the ratio of gene *n* expression in a sample under study to an average level in the control group; *ARR* (*activator/repressor role*) is a Boolean value that depends on the function of this gene product in pathway *p*. ARRs are Boolean values defined as follows: −1 when product of gene *n* inhibits *p*; 1 when product of *n* activates *p*; 0 when product of *n* has an ambiguous or unclear role in the pathway; 0.5 or −0.5, when the product of *n* is an activator or an inhibitor of *p*, respectively. As the reference gene expression profile, we used the artificial gene expression profile obtained by gene-by-gene averaging of all gene expression data in the cohort under investigation.

### 4.7. Statistical Analysis

ROC AUC value and *t*-test *p*-value were used as the measures of biomarker quality in comparisons of LGG vs. GBM, and for discriminating GBM molecular subtypes. *p*-values were adjusted according to Benjamini–Hochberg false discovery rate (FDR) method.

Overall survival was assessed by Kaplan–Meyer analysis; the statistical significance of differences was measured by log-rank test *p*-value after Benjamini–Hochberg FDR correction. Since only one of the three datasets has progression free survival (PFS) data, PFS was not investigated in this study.

Hazard ratios (HR) were calculated in univariate Cox model to assess differences in survival among the groups under comparison. The following simultaneous significance thresholds were used for high-quality biomarkers: FDR-adjusted *p*-value < 0.05, AUC > 0.7, HR confidence interval not including 1.

### 4.8. Gene Ontology Analysis

Gene Ontology (GO) classification of algorithmically deduced molecular pathways was performed with R package clusterProfiler [61]. To this end, each pathway was considered as a specific gene set, and statistically significantly enriched GO terms were detected (adjusted *p* < 0.05). Thus, each pathway obtained relevant GO terms (tags). Pathways could form functional groups by the similarities of the corresponding GO tags. For example, pathways with tag “cytokinesis” formed functional group “cytokinesis.” Since there were usually several GO tags identified per pathway, a pathway could be included in all relevant functional groups.

GO trees were built and visualized with Cytoscape and ClueGo [62,63]. For gene-based biomarkers, the obtained gene sets were used as the input data with the selected option “Functional analysis.” To characterize a set of pathway-based biomarkers in GO tree, we combined lists of GO tags (GO terms), which were previously obtained with ClusterProfiler for classification of the selected pathways. The combined list was used as input data for ClueGo, and the option “Preselected functions” was selected. We used the following GO tree visualization settings: network specificity = “global,” min level = 1, max level = 3, GO term grouping = TRUE, *p*-adjusted < 0.05.

### 4.9. Intersection Significance Tests

To test whether a given number of common items between the *n *(in this study *n* could be 2, 3, and 5) intersecting sets is significant, 10,000 random perturbations were performed according to [17,64]. In every comparison, *n* random subsets were taken and intersected. These random subsets were then intersected for each of 10,000 perturbations, and the *p*-value of intersection significance was calculated as a fraction of random perturbation cases giving an equal or higher number of common items compared to the experimentally observed samplings.

## Figures and Tables

**Table 1:** Best individual gene and gene-centric pathway expression biomarkers identified in the GBM–LGG comparisons.

Gene Biomarkers ^1^	Gene-Centric Pathway Biomarkers ^1,2^
***Up-regulated in GBM:*** *ANXA2*, *CHI3L1*, *EMP3*, *ESM1*, *FN1*, *GPX8*, *HSPA6*, *IGFBP2*, *ITGA5*, *LDHA*, *MMP9*, *PLAT*, *PLAUR*, *PLEK2*, *SERPINH1*, *SRPX2*, *STC1*, *TAGLN2*, *TNFRSF12A.* ***Up-regulated in LGG: *** *ALDH5A1*, *GABBR1*, *GLUD1*, *MMD2*, *NDRG2*, *SLC25A21-AS1*, *ZDHHC22.*	***Up-regulated in GBM:*** ANXA2-pw, CD44-pw, HRG-pw, ITGB3BP-pw, KLK4-pw, ME2-pw, MFAP2-pw, MFAP5-pw, PAK2-pw, PCOLCE-pw, PLG-pw, PRSS1-pw, PRSS2-pw, SDC4-pw, SERPINB8-pw, SERPINE2-pw, TACC3-pw. ***Up-regulated in LGG:*** gdhA-pw, KCND2-pw, KCNIP2-pw, PTPRJ-pw.

^1^ Case when there was both the best marker gene and the corresponding gene-centric pathway are underlined. ^2^ “pw” stands for “pathway.”

**Table 2:** Statistics of gene and pathway expression biomarkers distinguishing GBM molecular subtypes.

	**Mesenchymal vs. Proneural**	**Classical vs. Proneural**	**Mesenchymal vs. Classical**
**Gene biomarkers**	32	4	440
**Gene-centric pathway biomarkers**	283	0	254

**Table 3:** Best individual gene and gene-centric pathway biomarkers identified for the comparisons of GBM molecular subtypes.

	Mesenchymal vs. Proneural	Classical vs. Proneural	Mesenchymal vs. Classical
**Gene biomarkers**	-	*-*	***Upregulated in mesenchymal:*** *CD14*, *EVA1A*, *FAM20A*, *MMP19*, *PLAUR.* ***Upregulated in classical:*** *ACSBG1*, *C2orf72*, *ELOVL2*, *ELOVL2-AS1*, *FGFR3*, *HEPACAM*, *MYO10*, *NPAS3*, *PTPRA*, *SLC24A3*
**Gene-centric pathway biomarkers ^1^**	-	-	***Upregulated in mesenchymal:*** CD180-pw, CD300E-pw, MIR100-pw, MIR99A-pw

^1^ “pw” stands for “pathway.”

**Table 4:** Number of included GBM samples of different molecular subtypes according to [52].

Database/Subtype	Mesenchymal	Proneural	Classical
TCGA	50	46	57
CGGA_325	34	15	49
CGGA_693	55	78	57

## Data Availability

All data which were generated in the study are contained within the article or supplementary material.

## Supplementary Materials

The following supporting information can be downloaded at: https://www.mdpi.com/article/10.3390/ijms23137330/s1.
