# Supplementary material for: Next-Generation Grade and Survival Expression Biomarkers of Human Gliomas Based on Algorithmically Reconstructed Molecular Pathways
Source: Int J Mol Sci. 2022 Jun 30;23(13):7330. doi: 10.3390/ijms23137330 (PMC9266372; doi:10.3390/ijms23137330)
Supplement: Supplementary file 1 [file ijms-23-07330-s001.zip › ijms-1784843-supplementary.pdf]

International Journal of *Molecular Sciences*

MDPI

Supplementary Figure S1. Gene Ontology tree (from null to third level) for genes linked with unfavorable prognosis in LGG with un-methylated *MGMT*(A), favorable prognosis in LGG with un-methylated *MGMT*(B), unfavorable prognosis in LGG with methylated *MGMT*(C), favorable prognosis in LGG with methylated *MGMT*(D), unfavorable prognosis in LGG with *IDH* mutation(E). Bold red arrow points to «biological process» term that is a root node (null level of GO tree). Small grey nodes are parent nodes, connecting root node with significant terms of third level. Size of color nodes reflects adjusted p-value. Each term group marked with a color, if a term belongs to several groups, it gets corresponding sectors.

Supplementary Figure S2. Gene Ontology tree (from null until third level) built by pathway functional groups, linked with unfavorable prognosis in LGG with un-methylated *MGMT*. Bold red arrow points to «biological process» term that is a root node (null level of GO tree). Small grey nodes are parent nodes, connecting root node with significant terms of third level. Size of color node reflects percent share of gene-centric pathways, relevant to a GO group.

Supplementary Figure S3. Gene Ontology tree (from null until third level) built by pathway functional groups, linked with unfavorable prognosis in LGG with methylated *MGMT*. Bold red arrow points to «biological process» term that is a root node (null level of GO tree). Small grey nodes are parent nodes, connecting root node with significant terms of third level. Size of color node reflects percent share of gene-centric pathways, relevant to a GO group.

Supplementary Figure S4. Gene Ontology tree (from null until third level) built by pathway functional groups, linked with favorable prognosis in LGG with methylated *MGMT*. Bold red arrow points to «biological process» term that is a root node (null level of GO tree). Small grey nodes are parent nodes, connecting root node with significant terms of third level. Size of color node reflects percent share of gene-centric pathways, relevant to a GO group.

Supplementary Figure S5. Gene Ontology tree (from null until third level) built by pathway functional groups, linked with unfavorable prognosis in LGG with *IDH* mutation. Bold red arrow points to «biological process» term that is a root node (null level of GO tree). Small grey nodes are parent nodes, connecting root node with significant terms of third level. Size of color node reflects percent share of gene-centric pathways, relevant to a GO group.

Supplementary Figure S6. Gene Ontology tree (from null until third level) built by pathway functional groups, linked with favorable prognosis in LGG with *IDH* mutation. Bold red arrow points to «biological process» term that is a root node (null level of GO tree). Small grey nodes are parent nodes, connecting root node with significant terms of third level. Size of color node reflects percent share of gene-centric pathways, relevant to a GO group.

Supplementary Figure S7. Gene Ontology tree (from null until third level) built by pathway functional groups, linked with unfavorable prognosis in LGG with wild type *IDH*. Bold red arrow points to «biological process» term that is a root node (null level of GO tree). Small grey nodes are parent nodes, connecting root node with significant terms of third level. Size of color node reflects percent share of gene-centric pathways, relevant to a GO group.

Supplementary Figure S8. Gene Ontology tree (from null until third level) built by pathway functional groups, linked with favorable prognosis in LGG with wild type *IDH*. Bold red arrow points to «biological process» term that is a root node (null level of GO tree). Small grey nodes are parent nodes, connecting root node with significant terms of third level. Size of color node reflects percent share of gene-centric pathways, relevant to a GO group.

Supplementary Table S1. Significant gene-based OS biomarkers associated with favorable and unfavorable prognoses in general LGG cohort and subgroups: LGG with methylated *MGMT*, un-methylated *MGMT*, *IDH* mutation.

Supplementary Table S2. Gene Ontology terms for gene-based OS biomarkers associated with favorable and unfavorable prognoses in general LGG cohort and subgroups: LGG with methylated

MGMT, un-methylated *MGMT*, *IDH* mutation. Analysis was performed with Cytoscape+ClueGO for all levels of GO tree.

Supplementary Table S3. Significant gene-centric pathway-based OS biomarkers associated with favorable and unfavorable prognoses in general LGG cohort and subgroups: LGG with methylated *MGMT*, un-methylated *MGMT*, *IDH* mutation, *IDH* wild type.

Supplementary Table S4. Gene Ontology terms for significant gene-centric pathway-based OS biomarkers associated with favorable and unfavorable prognoses in general LGG cohort and subgroups: LGG with methylated *MGMT*, un-methylated *MGMT*, *IDH* mutation, *IDH* wild type.

Supplementary Table S5. AUC and t-test p-value for comparisons LGG vs GBM, and GBM molecular subtypes against each other.
