# Supplementary figures and images for: Next-Generation Grade and Survival Expression Biomarkers of Human Gliomas Based on Algorithmically Reconstructed Molecular Pathways
Source: Int J Mol Sci. 2022 Jun 30;23(13):7330. doi: 10.3390/ijms23137330 (PMC9266372; doi:10.3390/ijms23137330)

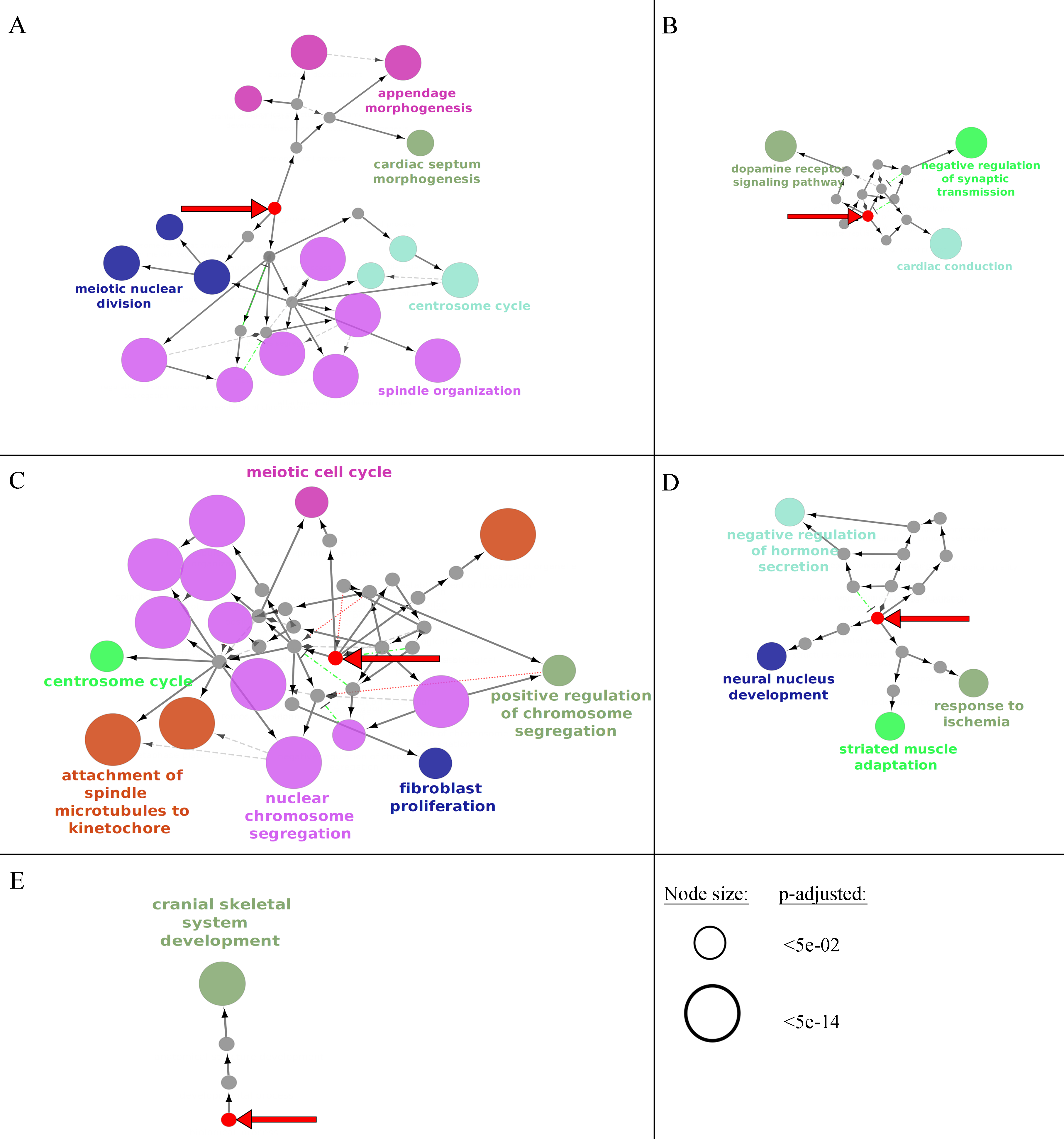

Supplement: Supplementary file 1 [file ijms-23-07330-s001.zip › Supplementary Figure S1.tif]

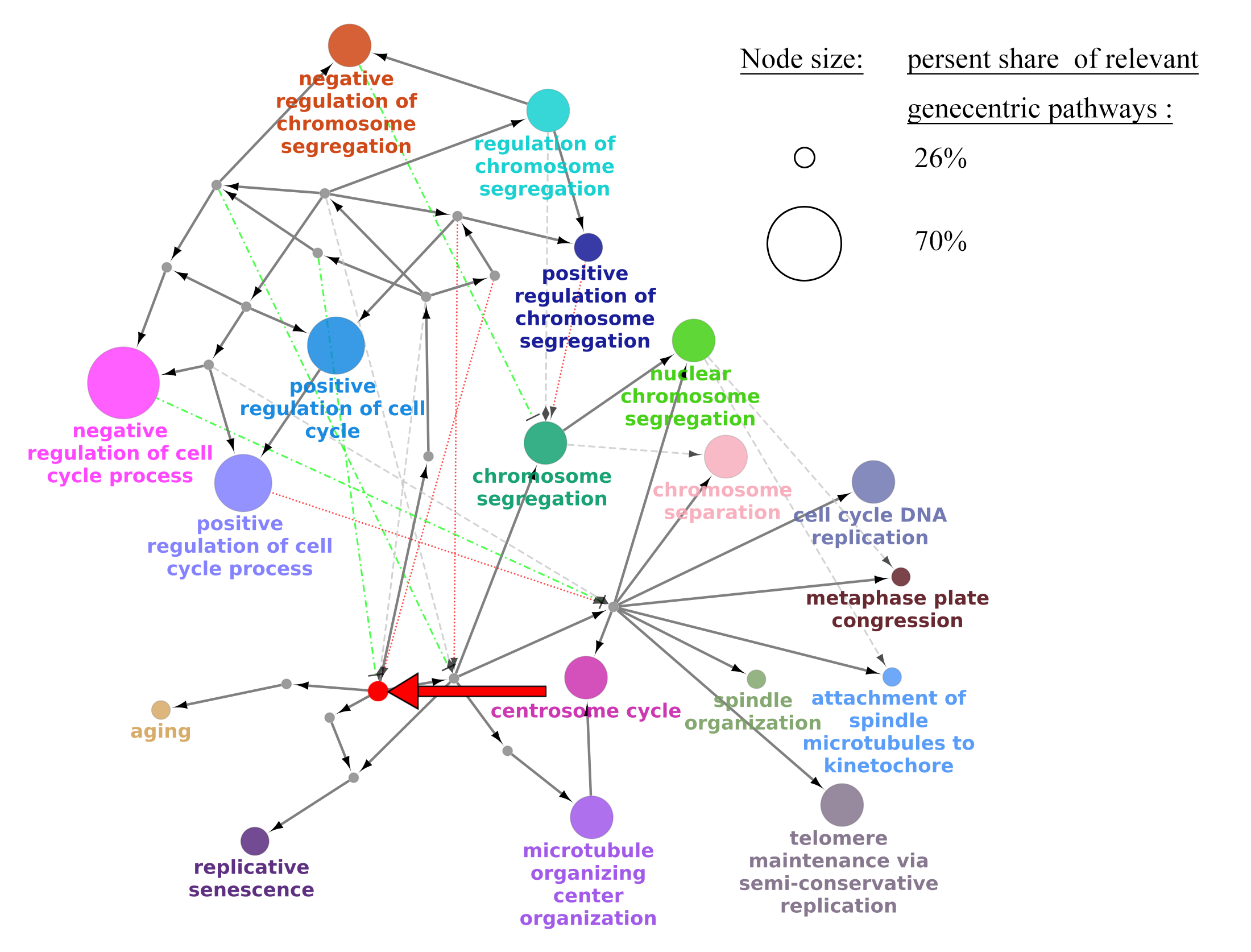

Supplement: Supplementary file 1 [file ijms-23-07330-s001.zip › Supplementary Figure S2.png]

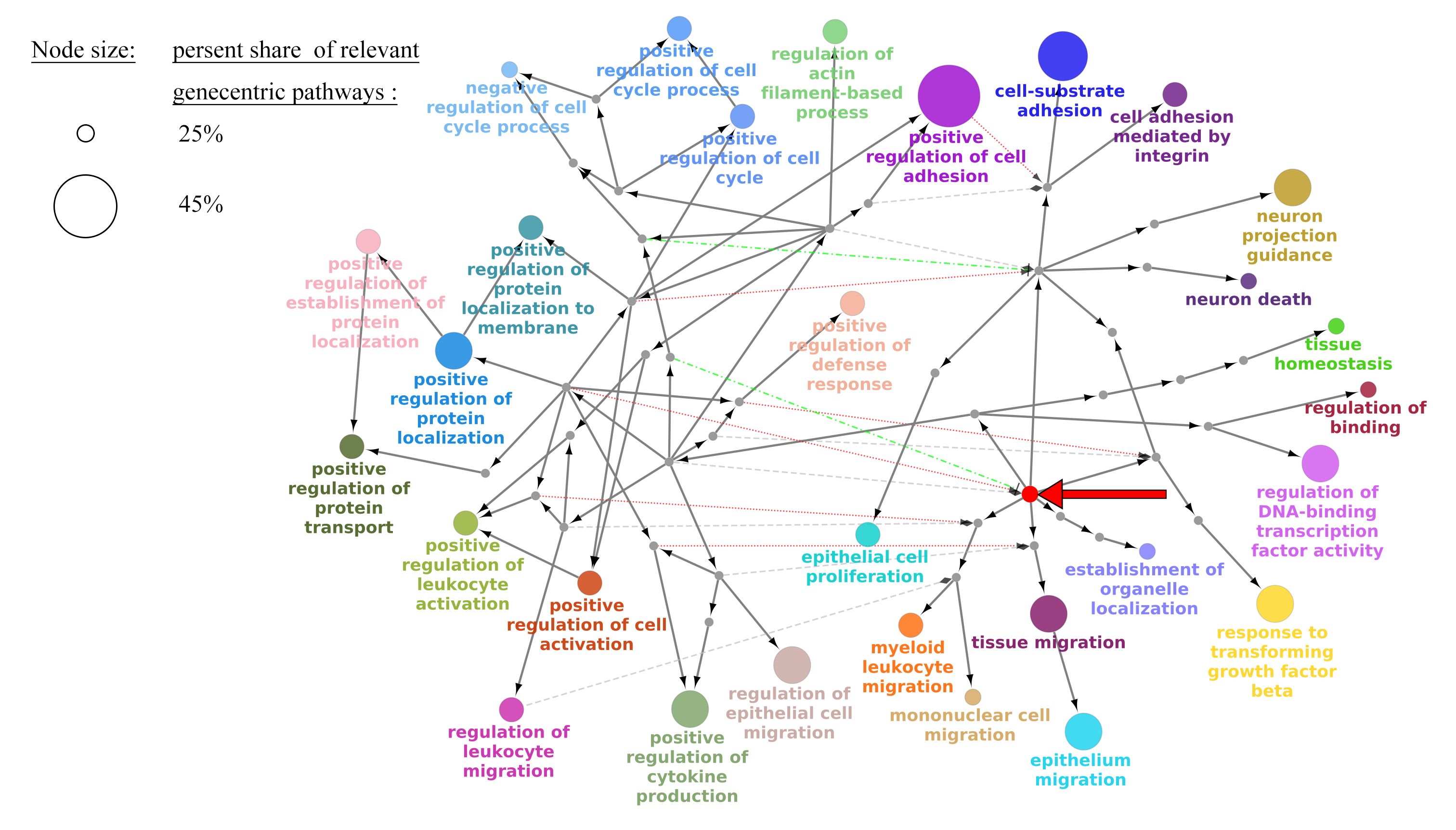

Supplement: Supplementary file 1 [file ijms-23-07330-s001.zip › Supplementary Figure S3.png]

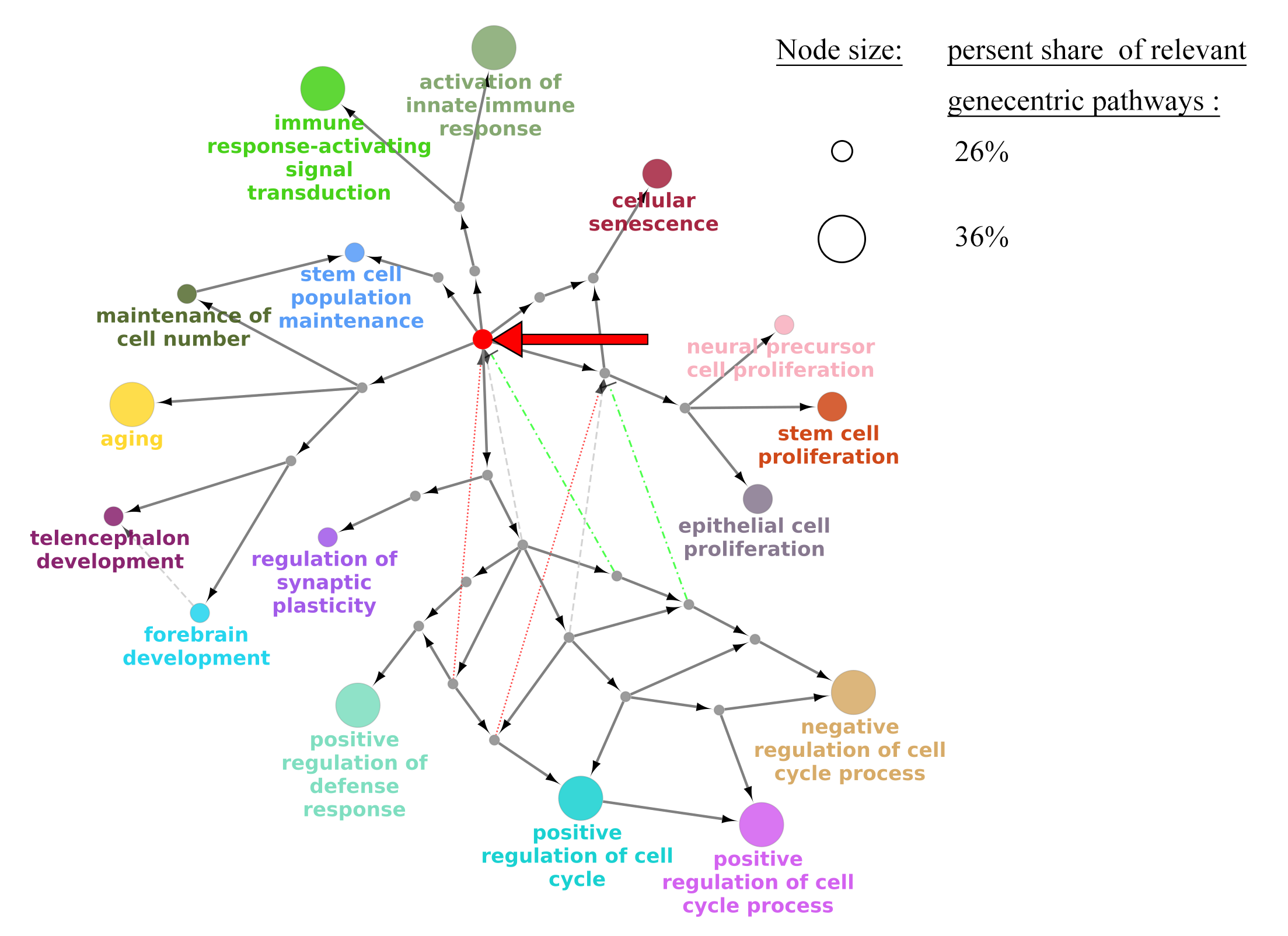

Supplement: Supplementary file 1 [file ijms-23-07330-s001.zip › Supplementary Figure S4.png]

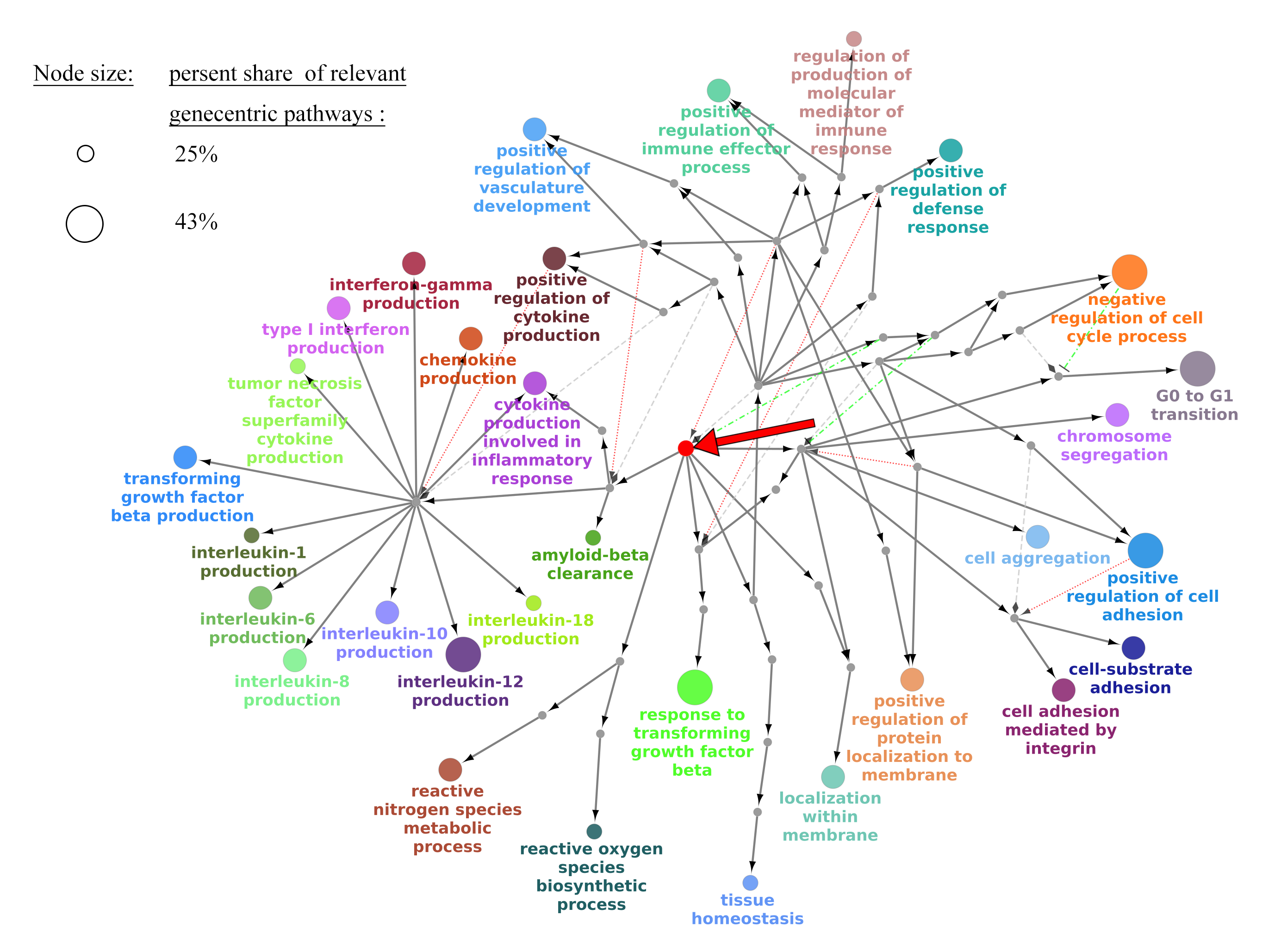

Supplement: Supplementary file 1 [file ijms-23-07330-s001.zip › Supplementary Figure S5.png]

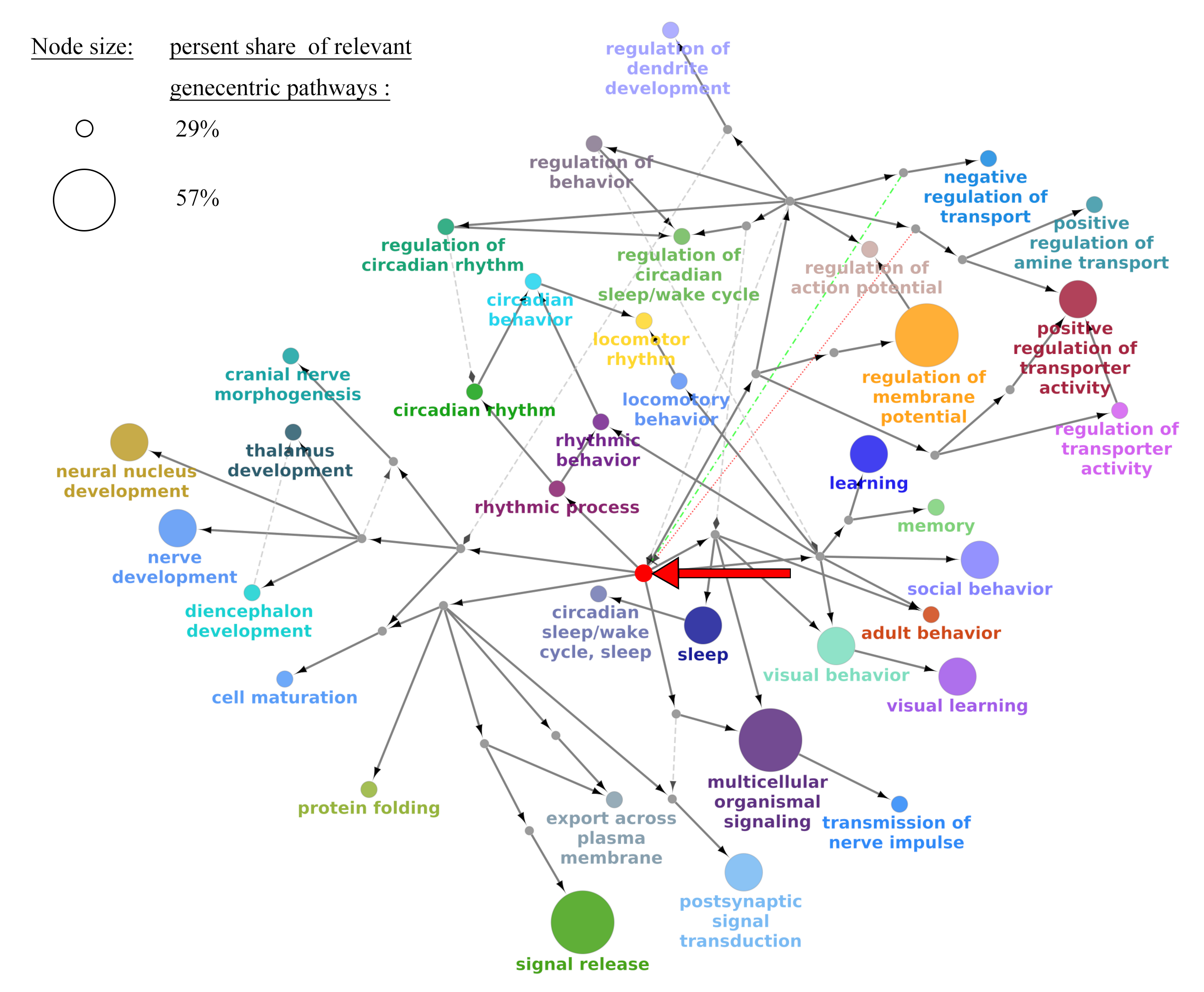

Supplement: Supplementary file 1 [file ijms-23-07330-s001.zip › Supplementary Figure S6.png]

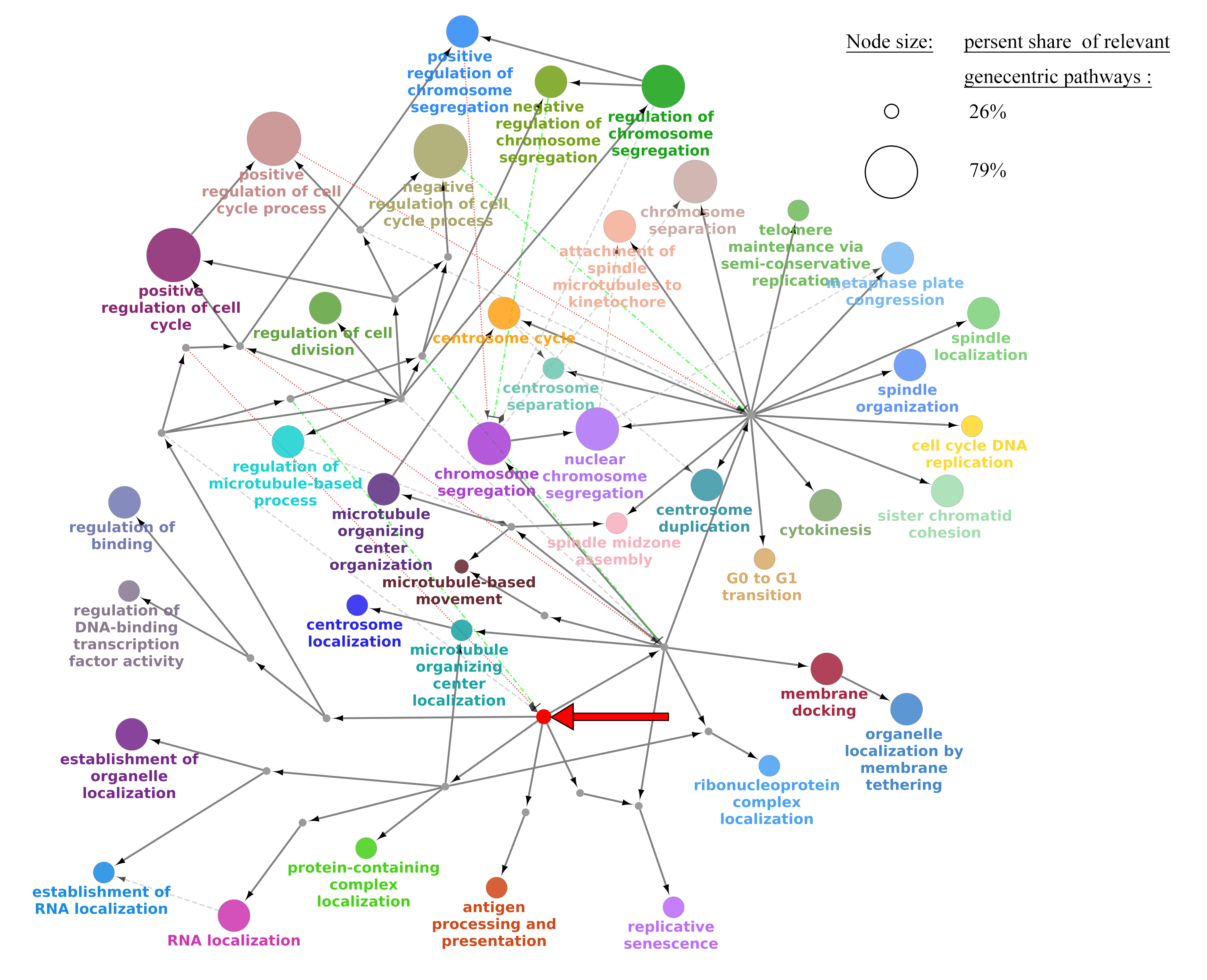

Supplement: Supplementary file 1 [file ijms-23-07330-s001.zip › Supplementary Figure S7.png]

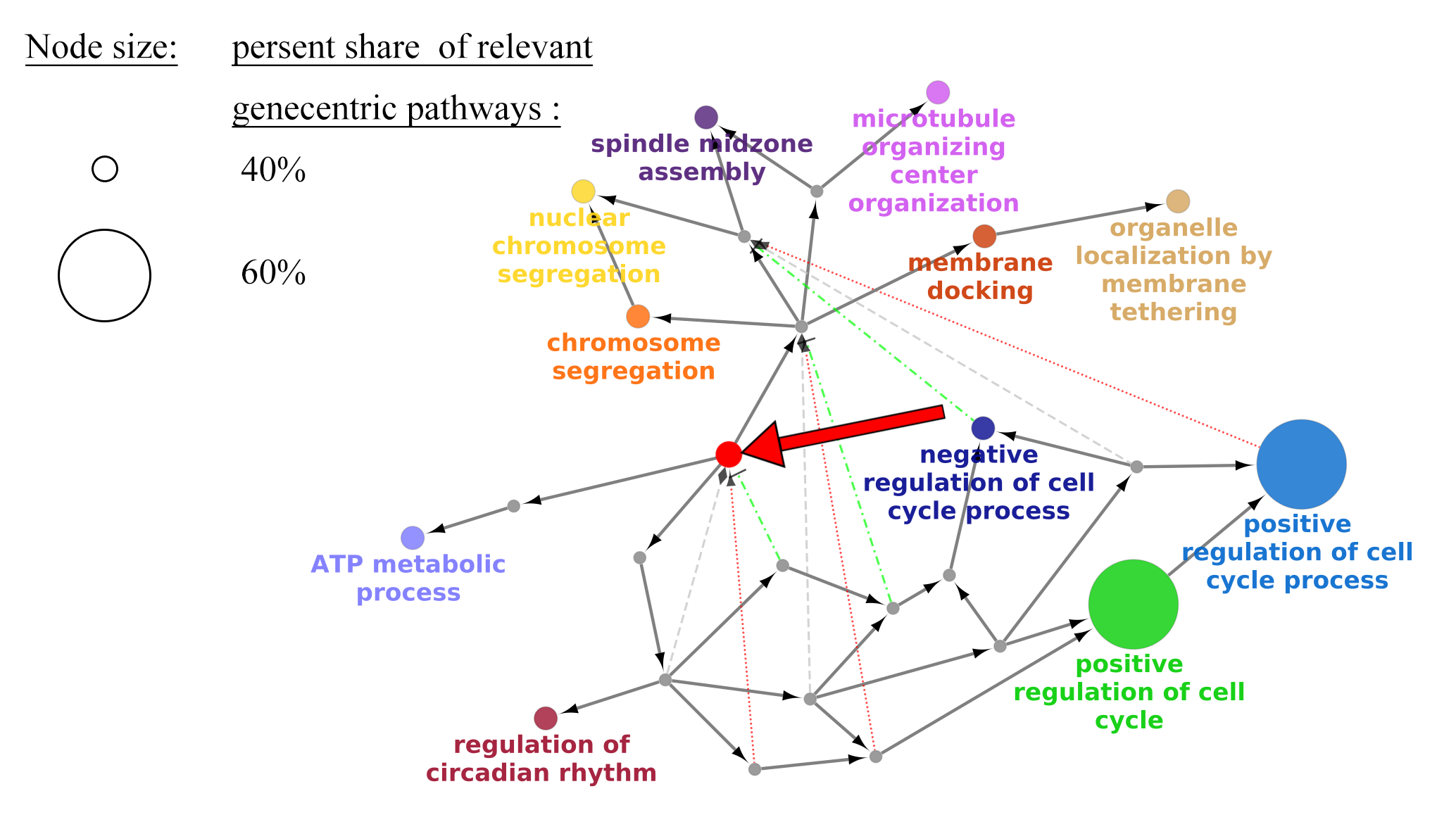

Supplement: Supplementary file 1 [file ijms-23-07330-s001.zip › Supplementary Figure S8.png]
